# Supplementary material for: Rational Design of HER2-Targeted Combination Therapies to Reverse Drug Resistance in Fibroblast-Protected HER2+ Breast Cancer Cells
Source: Cell Mol Bioeng. 2024 Oct 11;17(5):491–506. doi: 10.1007/s12195-024-00823-0 (PMC11538110; doi:10.1007/s12195-024-00823-0)
Supplement: Supplementary file 5 — Supplementary file5 (PDF 218 KB) [file 12195_2024_823_MOESM5_ESM.pdf]

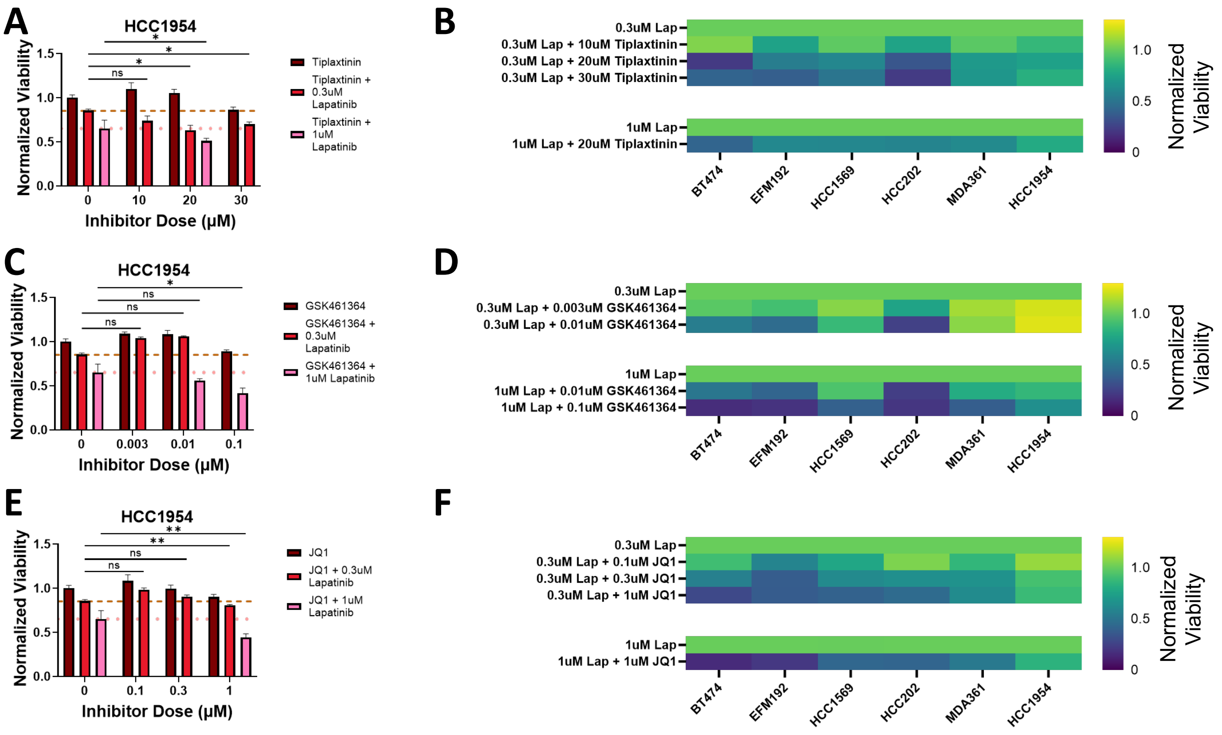

Figure S1: Fibroblast-insensitive HCC1954 cells are less responsive to combination therapies compared to fibroblast-protected cell lines.

(**A**) High doses of tiplaxtinin further reduces viability of HCC1954 cells treated with fibroblast-conditioned medium compared to lapatinib alone. Red and pink lines indicate viability in lapatinib-treated condition. Viability is normalized to untreated condition. (**B**) Response to tiplaxtinin (PAI1i) combination therapy for fibroblast-protected (BT474, EFM192, HCC1569, HCC202, MDA361) and fibroblast-insensitive (HCC1954) cells. Viability for combination therapies is normalized to lapatinib monotherapy. (**C**) HCC1954 response to GSK461364 (PLK1i) combination therapy is dose-dependent. (**D**) Viability of HER2+ breast cancer cell lines after treatment with GSK461364 combination therapy. Viability is normalized to lapatinib monotherapy. (**E**) Inhibition of PAI1 with JQ1 reduces HCC1954 viability compared to lapatinib alone. (**F**) Response of fibroblast-protected cell lines and fibroblast-insensitive HCC1954 cells to JQ1 combination therapy. *: p<0.05, **: p<0.01.

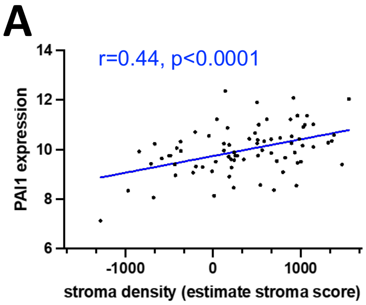

Fig. S2: PAI1 expression positively correlates with stroma score in independent dataset

(A) PAI1 expression in breast cancer patient data from The Cancer Genome Atlas (n=78) correlates with estimated stroma score. Pearson correlation coefficient $p<0.05$.
